# Effects of Musical Classes on Motor Creativity According to Age, Sex, and Weight Status in Young Students: A Music-Oriented versus Conventional Education Plan

**DOI:** 10.3390/children10020200

**Published:** 2023-01-20

**Authors:** Athos Trecroci, Gabriele Signorini, Raffaele Scurati, Dario Colella, Gaetano Raiola, Marta Rigon, Pietro Luigi Invernizzi

**Affiliations:** 1 Department of Biomedical Sciences for Health, Università degli Studi di Milano, 20133 Milano, Italy; 2 Department of Biological and Environmental Sciences and Technologies, University of Salento, 73100 Lecce, Italy; 3 Department of Political and Social Sciences, University of Salerno, 84084 Fisciano, Italy

**Keywords:** physical education, physical activity, motor skills, physical literacy, fitness, health, motor competence

## Abstract

Motor creativity can be influenced by the specificity of the school–class environments (music-based education plans) and individual characteristics. This study aimed to investigate the effects of music oriented and conventional education plans on rhythmic perceptive capacity, motor creativity, and skill- and health-related fitness components in young students according to age, sex, and weight status. One hundred sixty-three young Italian students from elementary (second and fourth grade) and middle school (sixth and eighth grade) were enrolled in the study according to their education plan (music oriented or conventional). All participants were tested for rhythmic perceptive capacity (Stambak’s test), motor creativity (Divergent Movement Ability test), skill-related (Körperkoordinationstest Für Kinder), and health-related (Multistage Fitness test) components. Individuals were also considered according to age (elementary and middle school), sex, and weight status. Significant age × education plan and sex × education plan interactions (*p* < 0.01) were found in motor creativity (locomotor and stability skills) and motor competence (balance and jumping-like activities). No significant weight status × education plan interaction was found. The predominant role of music in the music-oriented education plan appeared to foster the ability to enhance motor creativity in elementary and middle school students compared to the conventional plan. Moreover, music-oriented experience also seems relevant for expressing and exhibiting motor competence (i.e., balance) in relation to sex.

## 1. Introduction

Developing motor skills for well-being and appropriate movement skills can foster prolonged motor autonomy along with competence, motivation, confidence, sensitivity, and awareness of own individual endowment or potential (i.e., physical literacy). With this in mind, the “educational universe” represented by the school should pave the way for spreading this condition to children and adolescents who have the right to develop their personality, talent, and motor competence fully [1,2]. Specifically, children who have a high level of skill-related fitness (e.g., motor competence in the form of coordinative, agility and balance-related tasks) are more likely to have an active lifestyle and a better health-related fitness profile (i.e., cardiorespiratory endurance), reaching and maintaining a healthy weight and mind in their life spans. Motor competence is associated with motor creativity, which is an extension of it [3]. Three dimensions can be investigated in motor creativity: fluency, originality, and flexibility. Fluency refers to the ability to produce many motor solutions, while originality is the ability to generate new and unique solutions. Flexibility refers to the ability to generate solutions relevant to different ideas of movement categories [4].

Motor creativity can be emboldened through teaching approaches based on exploration and discovery processes within diverse environments (e.g., conventional or subject-oriented education plans based on art and music) where motor variability assumes a crucial role in producing personal and functional or expressive solutions (creative movement and thinking) linked to new, original, and relevant motor tasks [5]. For instance, it was previously stated that specific environmental characteristics (e.g., the presence of music) might put an individual in the condition to achieve their well-being by developing different areas (motor, cognitive, and affective) and expressing feelings and emotions (with their body) that would be hard to be exclusively expressed in verbal modes [6]. Suffice it to think of the coordination of rhythmic movement with an external rhythm (e.g., music oriented) that is relevant to motor, cognitive, and social behavior [7].

The ability to generate many varied motor solutions, in which motor creativity and skill-related fitness components are involved, is also influenced by an individual’s health-related fitness level, which, in turn, is limited by their weight status. Intuitively, an overweight status (e.g., presenting a high body mass index value) would put a child in a condition of an inferior motor competence [8,9,10] and cardiorespiratory endurance with a limited ability to move.

Music and creativity might be considered two sides of the same coin. Creativity may be rooted in areas of the brain associated with attention, planning, working memory, cognitive flexibility, and imagination, which could be influenced by musical stimuli (input). Indeed, regions most consistently implicated in creative musical behavior are not auditory but motor regions [11] as the primary motor cortex that contributes to musical creativity, also mediating the potential for creative motor action (output) [12]. Moreover, whether additional musical stimuli (e.g., undergoing a music-oriented education plan at school) influences premotor and frontal regions of the brain even depends on the age at which it is initiated and consolidated. For instance, early childhood (e.g., from 4 to 7 years of age) is particularly sensitive to this type of stimulation [13,14]. However, besides particular critical periods, greater or lesser experiences concerning the same environmental stimulation (additional musical class attendance) take part in the process linked to music, creativity, and movement. It would be relevant to investigate how music, creativity, and movement are interconnected in young school-attending individuals according to their age, sex, and weight status.

Therefore, the main purpose of the present study was to investigate the effects of both music-oriented (M-O) and conventional (C) education plans on rhythmic perceptive capacity, motor creativity, and skill- and health-related fitness components in young students according to their age, sex, and weight status. The results of this study could provide helpful insights to plan even more successful schooling interventions supporting the mutual interaction between environment and individual physical and psychomotor development as a dynamic multilevel system contemplating sensory perception, thinking, and action.

## 2. Materials and Methods

### 2.1. Participants

One hundred sixty-three Italian elementary (second and fourth grade) and middle school (sixth and eighth grade) students voluntarily participated in the study. They were selected from schools employing either M-O or C. A comprehensive description of the participants’ characteristics is reported in Table 1. M-O and C differed in relation to the music hours on the total education hours for elementary and middle school students. The total education hours of both C and M-O were 31 h and 34 h per week for elementary and middle school students, respectively. For C, their corresponding music hours consisted of 1 h (60 min) and 2 h (120 min) per week, respectively. Conversely, for M-O, the total musical classes consisted of 140 min (60 min + 80 min) and 280 min (120 min + 160 min), respectively. The additional music hours were divided as follows: (i) 40 min each of instrumental and solfege lessons in elementary school students; (ii) 60 min each of solfege lessons and ensemble music, along with 40 min of instrumental lessons in middle school students. Overall, 7% and 15% were the relative contributions of the music hours over the total for M-O in elementary and middle school students, respectively. Conversely, for C, the music hours were 3% and 6% of the total education hours in elementary and middle school students, respectively.

All participants and their parents were informed about the purpose and experimental protocol of the study. Parents or legal guardians provided written informed consent before the investigation. Under the Declaration of Helsinki, the study was approved by the Ethics Committee of the University of Milan (approval number 18/22).

### 2.2. Procedures

**Figure 1:**
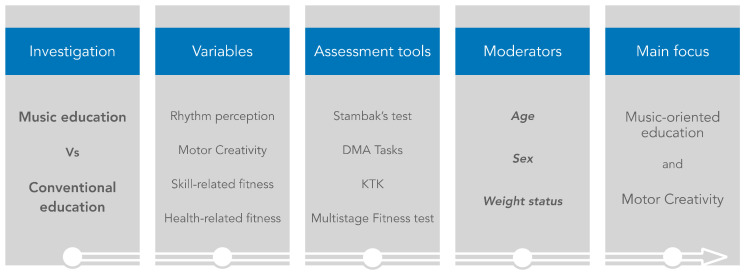
Schematic representation of the experimental concept. (DMA) Divergent Movement Ability. (KFK) Körperkoordinationstest für Kinder.

This study was conducted based on a cross-sectional design of which the experimental concept is shown in Figure 1. The experimental protocol was carried out within morning school hours using spaces granted by the schools, including two gymnasiums and a courtyard. Anthropometric measures of height and weight were recorded to the nearest 0.1 cm with a standing stadiometer (Seca 217, Basel, Switzerland), and body mass was measured to the nearest 0.1 Kg with a high-precision mechanical scale (Seca 877, Basel, Switzerland). Then, body mass index (BMI) was calculated as the ratio of body mass to height squared. The obtained BMI values were compared with the corresponding percentiles from previous normative data on growth [15], classifying individuals as healthy weight, overweight, or obese [16]. For convenience, participants exhibiting a healthy weight were deemed “*normal weight*”, while those overweight were deemed “*overweight*”. Before testing, all participants were familiarized with all experimental procedures. The familiarization process included testing sessions for rhythmic perceptive capacity, motor creativity, and skill- and health-related performance, separated by a 24 h interval among them. Seventy-two hours after the familiarization, two experimental assessment sessions were accomplished during the morning school hours, one week apart from each other.

### 2.3. Rhythmic Perceptive Capacity

**Figure 2:**
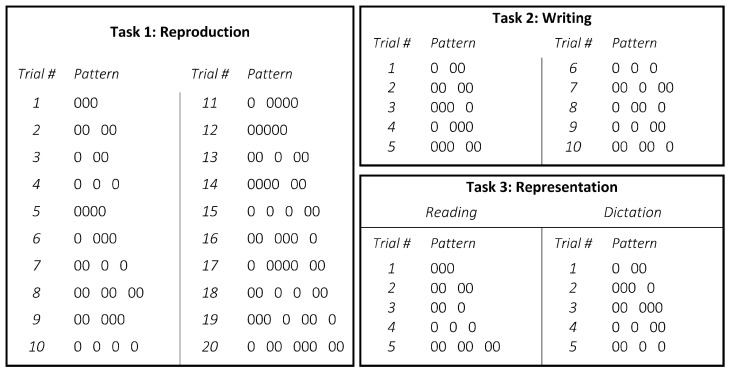
Reproduction, writing, and representation of the tasks of Stambak’s test. Circles correspond to beats and spaces between circles to silence.

Stambak’s test [17,18] measures an individual’s level of spatio-temporal structuring by testing the rhythmic ability on multiple levels: (i) replication of temporal structures (Figure 2, Task 1: the examiner plays twenty rhythmic sequences in which each circle corresponds to a beat and each space between the circles corresponds to silence. The child is requested to reproduce it in the way he or she thought was most similar); (ii) symbolization of spatial structures (Figure 2, Task 2: the examiner shows—one by one—ten rhythmic structures. Immediately after showing a structure for a few seconds, the examiner hid it from the participant. Once memorized the structure, the participant reproduces graphically each of them as closely as possible); (iii) symbolization of temporal structures (Figure 2, Task 3: the examiner reproduces the rhythmic structures by tapping. After listening, the participant reproduces graphically the sequence attributing appropriate circles and spaces). In each task, after two consecutive incorrect sequences, the test ends. One point is awarded for each successful structure, and the points obtained for each level are added.

### 2.4. Motor Creativity Assessment

The Divergent Movement Ability (DMA) test was employed to examine the creativity of school-aged children [19,20]. The DMA measures the fluency and flexibility dimensions of creativity for each of the following fundamental movement skills: (i) locomotor, for which an individual is asked to use the available tools (e.g., two mattresses of different sizes, four cones arranged diagonally, a suspended rope, and a hula hoop supported by three supports) encouraging them to create as many movements as possible; (ii) stability, for which an individual is free to take on different poses (with their body parts) within an area with a 45.5 cm high bench placed in the middle; (iii) manipulation, for which an individual handles a ball (23 cm diameter) within an area (3 m × 4.5 m) delimitated by cones (on three sides) and a wall (on the remaining side).

Participants were tested on their ability to manipulate the ball. They were constantly motivated to provide as many solutions as possible without feedback about their performance.

For each skill, two time trials were given with the following arrangements: 90 s for each trial with a 60-s resting period in between. Then, 120 s of rest were given between each typology of fundamental movement skill.

All trials were recorded using a video camera (Canon Legria HF-R17), and the derived video footage was independently analyzed a posteriori by two observers. The agreement of the two observers was computed as previously reported [21] with a 95% and 82% agreement level for intra-observer and inter-observer evaluations, respectively. Flexibility was calculated by summing up the number of motor patterns provided (and recorded) on each different skill. Fluency was assessed by counting the number of changes in the theme of the tasks performed, scheduled, or added to the evaluation sheet. The number of responses produced during the two 90-s sessions were summed, each motor pattern and their respective changes representing “different” responses. The total values of each fundamental movement skill represented the participant’s ability to produce divergent movement solutions.

### 2.5. Skill-Related Fitness Assessment

The Körperkoordinationstest Für Kinder (KTK) was administered to test motor coordination as commonly used in children and adolescents [22]. It is a valid and reliable test having a simple and relatively low time-consuming execution. KTK consists of four sub-tests: (i) walking backwards along three wooden beams of progressively smaller widths (6 cm, 4.5 cm, and 3 cm). The number of steps performed on the beam, excluding the first one, is counted. Three trials were allowed for each beam; (ii) one-leg hopping for the height of an obstacle composed of foam panels (5 cm high × 60 cm long × 20 cm wide). An individual is requested to perform two jumps before approaching the obstacle and two jumps after passing it, executed on each leg. Three, two, or one points can be awarded in relation to the number of successful trials. A final score is obtained by summing up the points obtained from each leg; (iii) moving sideways on square-shaped wooden tiles (20 cm × 20 cm). It comprises two trials lasting 20 s each with a 10-s resting period in between. An individual must perform as many lateral displacements as possible by moving from one tile to the other. The total number of lateral displacements counted within the two trials and the number of tile relocations are considered; (iv) jumping laterally (side by side) for 15 s over a wooden bar placed on the floor. Two trials were allowed, and the total number of jumps was summed up. A 10-s resting period interspersed the two trials. The scores obtained in each sub-test, namely raw values, were noted in an appropriate sheet, normalized through dedicated nomograms for age and sex, and summed up to obtain the motor quotient (MQ).

### 2.6. Health-Related Fitness Assessment

The Multi-Stage Fitness test was administered in an indoor gym to examine the health-related fitness profile reflected by cardiorespiratory endurance [23]. This incremental running test to exhaustion requires an individual to run between two lines set 20 m apart at a specific pace following pre-recorded tones at appropriate intervals. The velocity was 8.5 km·h^−1^ for the first minute, which increased by 0.5 km·h^−1^ every minute thereafter. The test terminates when the subject can no longer maintain the required velocity and reach the line on time. The total distance achieved was recorded and considered the main outcome.

### 2.7. Statistical Analysis

The Shapiro–Wilk’s test was conducted to verify data distribution. Test–retest reliability was performed using the intraclass correlation coefficient (ICC). A two-way analysis of variance was used to detect possible simple (age/sex/weight status × educational plan interactions) and main effects of age/sex/weight status and educational plan. The proportion of variance attributable to each effect and the magnitude of the difference were estimated by the partial eta-squared method (ɳ_p_^2^; small = 0.02, medium = 0.13, large = 0.26). Significance was set with *p* ≤ 0.05. The analysis was performed using the IBM SPSS^®^ Statistics software (v. 21, New York, NY, USA).

## 3. Results

ICC values showed moderate reliability for Stambak’s test (ICC = 0.700), good reliability for DMA (locomotor, ICC = 0.905; stability, ICC = 0.888; manipulative, ICC = 0.811) and KTK subtests (walking backwards, ICC = 0.889; hopping for height, ICC = 0.862; moving sideways, ICC = 0.863; jumping laterally, ICC = 0.841), MQ score (ICC = 0.823), and excellent reliability Multistage Fitness test (ICC = 0.961). All data are presented in Table 2.

**Figure 3:**
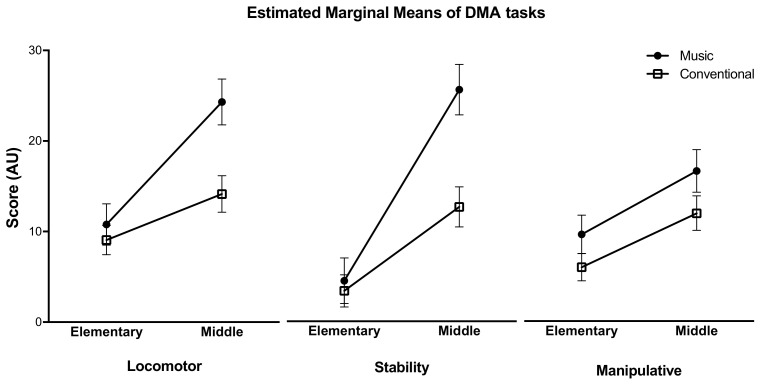
Estimated marginal means (output) of the locomotor, stability, and manipulative skills of the Divergent Movement Ability test in elementary and middle school students.

Regarding analyses by age, significant main effects of age and education plan were found only in Stambak’s test (*p* < 0.0001, ɳ_p_^2^ = 0.345). Meanwhile, a significant interaction (age × education plan) was observed in the DMA locomotor (F(_1,148_) = 15.212, *p* < 0.0001, ɳ_p_^2^ = 0.087) and stability (F(_1,148_)= 24.719, *p* < 0.0001, ɳ_p_^2^ = 0.135) tasks (Figure 3). For the manipulative tasks, only significant main effects of age and education plan (M-O and C) were observed (*p* < 0.0001, ɳ_p_^2^ = 0.097). As music and creativity are highly expected to have connections with each other, and since interactions by age due to music and conventional plans application were found only in DMA tasks, we reported graphically only the estimated marginal means of the DMA tasks (Figure 3).

Concerning the KTK test, there was only a significant interaction (age x education plan) in the jumping laterally sub-test (F(_1,139_) = 6.907, *p* = 0.010, ɳ_p_^2^ = 0.047), while walking backwards (*p* = 0.006, ɳ_p_^2^ = 0.053), moving sideways (*p* = 0.001, ɳ_p_^2^ = 0.134), and MQ scores (*p* < 0.0001, ɳ_p_^2^ = 0.125) presented only a significant main effect of age (elementary and middle school age). Moreover, a significant (*p* < 0.0001, ɳ_p_^2^ = 0.363) main effect of age was detected in the Multistage Fitness test.

**Figure 4:**
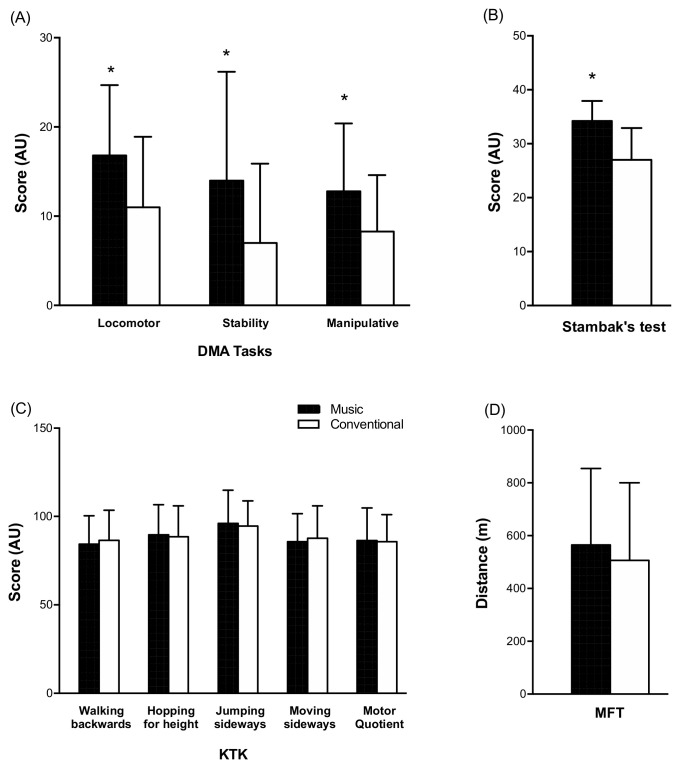
The main effect of the education plan on the measured variables. (**A**) Divergent Movement Ability tasks. (**B**) Stambak’s Test. (**C**) Körperkoordinationstest Für Kinder. (**D**) Multistage Fitness Test. * = different than conventional (*p* < 0.05).

In regard to the sex-related analysis, a significant (*p* < 0.0001, ɳ_p_^2^ = 0.345) effect of the education plan was observed in Stambak’s test (Figure 4). Similarly, DMA showed a significant (*p* < 0.0001) main effect of the education plan for locomotor, stability, and manipulative skills (Locomotor ɳ_p_^2^ = 0.108, Stability ɳ_p_^2^ = 0.092, Manipulative ɳ_p_^2^ = 0.085; Figure 4), with the latter also presenting a significant (*p* = 0.048, ɳ_p_^2^ = 0.024) main effect of sex. KTK presented significant interaction (sex × education plan) in the walking backward (F(_1,139_)= 6.305, *p* = 0.013, ɳ_p_^2^ = 0.043), hopping for height (F(_1,139_) = 7.250 *p* = 0.008, ɳ_p_^2^ = 0.050), and MQ scores (F(_1,139_) = 5.625, *p* = 0.019, ɳ_p_^2^ = 0.001). For jumping sideways, only a significant main effect of sex was found (*p* < 0.0001, ɳ_p_^2^ = 0.165). Neither interaction nor main effects were observed in the Multistage Fitness test (*p* > 0.05). In regard to weight status in Stambak’s test, it was only observed as a significant (*p* < 0.0001, ɳ_p_^2^ = 0.345) main effect of the education plan. In the DMA test, no interaction significant main effects of weight status and education plan were found in the locomotor (*p* < 0.0001, ɳ_p_^2^ = 0.103 and *p* = 0.004, ɳ_p_^2^ = 0.051), stability (*p* < 0.0001, ɳ_p_^2^ = 0.108 and, *p* = 0.014, ɳ_p_^2^ = 0.037), and manipulative (*p* = 0.003, ɳ_p_^2^ = 0.054 and *p* = 0.009, ɳ_p_^2^ = 0.042), respectively. Conversely, neither interaction (weight status × education plan) nor significant main effects were found in the walking backwards (*p* = 0.005, ɳ_p_^2^ = 0.005), hopping for height (*p* < 0.0001, ɳ_p_^2^ = 0.098), jumping laterally (*p* < 0.0001, ɳ_p_^2^ = 0.143), MQ score (*p* < 0.0001, ɳ_p_^2^ = 0.121), and moving sideways (*p* = 0.034, ɳ_p_^2^ = 0.035) sections of the KTK test. In the Multistage Fitness test, only a significant main effect of weight status was observed (*p* < 0.0001, ɳ_p_^2^ = 0.120).

## 4. Discussion

The main findings of this study revealed that participants attending a music-oriented education plan presented higher creativity than those attending a conventional one (M-O versus C). Moreover, creativity changed according to the student’s age, with middle school students exhibiting significantly higher creativity for locomotor and stability tasks than their younger peers.

To the authors’ knowledge, this is the first study investigating how music, creativity, and movement are interconnected according to age, sex, and weight status in young students. In the DMA, the locomotor and stability tasks’ outcomes revealed that the M-O plans affected motor creativity depending on the specific expertise. After years of practice, middle school participants attending M-O plans developed higher motor creativity than their younger peers. It is worth mentioning that the number of music hours included in M-O plans significantly differed between elementary and middle school students, whose hours were doubling. However, most importantly, the motor creativity of the students following the M-O education plan was significantly higher than their peers following the C plan. Specifically, in the face of a ~130% difference in weekly music hours (60 min of C vs. 140 min of M-O for elementary-school and 120 min of C vs. 280 min of M-O for middle-school), DMA tasks resulted, in average, ~37 and ~71% higher in the M-O plan than the C plan for elementary and middle school students, respectively.

Specific environmental characteristics may put an individual in the condition to achieve their well-being by developing different areas of physical literacy (i.e., perceptive and psycho-motor) [24,25]. This condition would confirm the crucial role of music in enhancing creativity across a range of movements.

Rhythm perception capacity measured by Stambak’s test showed a significant main effect of age (in favor of middle school students) and education plan (in favor of M-O). Again, the former effect may be explained by the years of musical classes attended by old against young students in rhythm perception capacity. Practicing with musical instruments strongly influences an individual’s rhythmic ability [26]. Beyond that melodic competency, it would concur in justifying the observed main effect of the education plan. In this study, the higher amount of music hours of the M-O plan (~130% more than C) led to a better rhythm perception capacity of approximately more than ~27% of C.

In regard to KTK outcomes, the overall results indicate that, regardless of the education plan, elementary school students performed better than their middle school peers. It should be noted that KTK scores were normalized by age, so the current results should be interpreted as relative instead of absolute. Early adolescents undergo remarkable neurological and anthropometrical changes during their developmental phase that could lead to moving less tightly by different coordinative structures [27]. This phenomenon is called *adolescent awkwardness*. Conversely, in early childhood, individuals appear more sensitive to coordination stimuli, resulting in a *window of opportunity* to learn and develop skills [28]. Although speculative, the lower motor competence exhibited by middle school students compared to their elementary school peers might originate from a “perfect storm” represented by the product of negative and positive effects of the aforementioned *awkwardness phase* and *window of opportunity*. As the Multistage Fitness test predominantly assesses cardiorespiratory endurance, the physical superiority of the older participants is supported by their advanced physiological status compared to younger peers.

According to the present findings, it seems that the skill-related fitness (i.e., motor competence) measured by the KTK changed by sex with the M-O and C plans. Specifically, males and females attending the M-O plan presented fewer walking backwards (balance skills) differences than their peers attending C. In regard to balance skills only, a specific sex-related difference (in favor of females) in the same KTK sub-test was previously reported [29]. A recent systematic review [9] also supports this. According to the observed interaction, it appears that the gap in balance ability between sex would be mitigated in those individuals attending the M-O plan. However, due to the observational nature of the current study design, it would hardly attribute a role to music to explain the above findings. Hence, it would be desirable to plan ad hoc intervention studies to clarify the role of sex in attending or not attending music classes on balancing skills in young students.

In the hopping for height and MQ tests, sex differences were significantly higher in the M-O individuals than in the C peers. Music encourages spontaneous rhythmic coupling between sensory and motor systems [30] that might improve the internal cueing system, and then promote better rhythmic motor tasks (i.e., running start) when approaching the jump. It is worth noting that, in pathological conditions associated with a loss of the internal cueing system (i.e., Parkinson’s disease), rhythmic motor tasks (i.e., walking and speech production) are impaired [31]. An individual can maintain the continuity of the running start action while developing the horizontal kinetic energy into a hop without arresting the movement between the two phases (running start and hop). It might be speculated that individuals attending musical classes are advantaged when dealing with exercise presenting a rhythmic structure as hopping for height. Concerning MQ, the current results align with those already observed in the literature. Overall, motor competence is higher in males than in females [9], especially in locomotor tasks, which are predominant in KTK sub-tests [29]. Males outperformed females in jumping-like activities (i.e., jumping laterally and hopping for height) where strength level is the main contributor. Rhythm perception capacity measured by Stambak’s test did not show any differences between sex, but a main effect of the education plan was observed. Observed for age, this result agrees with supporting the practice of musical instruments to strongly influence an individual’s rhythmic capacity beyond that of melodic competency [26].

The current results linked to different weight status (normal weight vs. overweight) conditions showed a better performance of normal weight individuals against overweight in all tests except for Stambak’s test outcomes. It should be noted that the current test battery, including KTK, DMA tasks, and Multistage Fitness test, required all participants constantly moving in space. Instead, they remained seated during Stambak’s test execution. This agrees that an overweight condition alters how an individual can move [9,10]. Mostly, overweight individuals exhibit lower physical activity levels that impair their muscle fitness outcomes [32]. This condition occurs regardless of the type of plan (M-O and C). Perhaps, further aspects (i.e., physical education) would play a leading role in mediating the present results. For instance, although out of the scope of this study, the role of the teacher would become crucial to foster the affective/behavioral, physical, and cognitive domains without bolstering the obesity barrier [10]. Additionally, teachers aware of the possible psychomotor limitations of overweight primary and secondary school children could help provide a framework for more focused interventions [33]. Lastly, future studies should emphasize extracurricular activities to investigate their potential impact on motor development by age, sex, and weight status [34].

### Limitations

The present study has some limitations. First, the small number of participants, specifically the music-oriented middle school students, suggests caution in data interpretations. However, the results from the specific approach of this first study are encouraging and should be subject to verification in more extensive investigations.

Second is the lack of control of additional elements that could have influenced motor creativity. For example, some teaching styles used by music teachers (e.g., the exploration-, discovery-, and variation-based styles) are particularly effective in supporting the development of thinking and motor creativity. Moreover, further curricular and extracurricular subjects (e.g., drawing, foreign language, theatre, dance, and sport activities) might have stimulated students’ expressivity or divergent thinking. Further study should account for the observation or control of such possible variables.

Finally, the period in which the study was conducted (end of the school year, namely March to May) might represent another variable to consider in the future. It is possibly associated with more intense and fatiguing curricular activity for the students, compared to a less demanding requirement typical of the first part of the school year, and should be worthy of more attention.

## 5. Conclusions

This study showed that motor creativity (in locomotor and stability movement skills) presented higher in elementary and middle school students following an M-O education plan compared to C. The predominant role of music might foster the ability to generate many varied motor solutions in which motor creativity and skill-related fitness components are involved. Indeed, the coordination of rhythmic movement with an external rhythm (e.g., M-O) could be relevant for expressing and exhibiting motor competence, as observed in KTK. Although attending or not, the M-O plan seems moot to mediate the observed weight status (normal weight vs. overweight) outcomes, however, it might be desirable for young students to be involved in a physical education process characterized by rhythm-based didactic enrichment to nurture their creativity.

Future Directions

**Figure 5:**
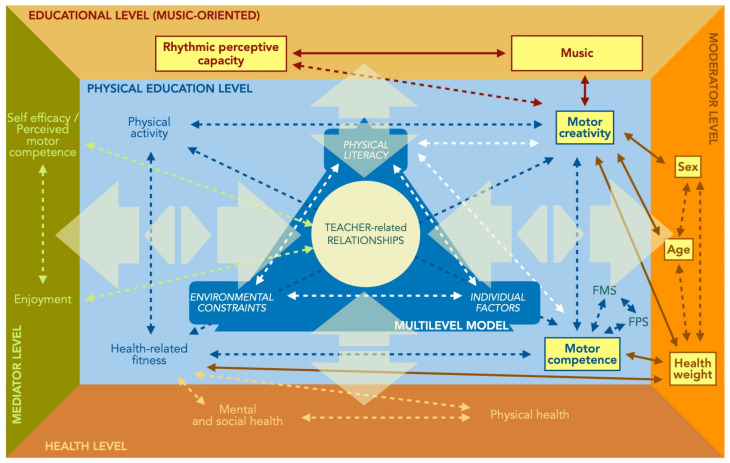
The framework of the ecological model in physical education. Yellow labels: core categories in the present study. Continuous lines: main detected effects. FMS = fundamental motor skills; FPS = fundamental play skills.

This study is part of a framework (Figure 5) aimed at considering all the factors that might increase or decrease the effects of children’s physical activity (general or specific) and the optimal development of individual potentials. Indeed, according to the socio-ecological models [35,36,37,38], children’s behaviors are the result of reciprocal environment interactions in a multilevel system including psychological mediators (enjoyment, self-efficacy, perceived motor competence), biological moderators (age, sex, health weight), and physical, mental, and psycho-social health, all of them pursued under educational aspects, such as teacher’s competence, teaching styles, and pedagogical sensitivity.

This study specifically considered the aspects related to motor creativity and music, focusing on their observation to better comprehend how the different factors interact, amplify, or inhibit motor creativity, starting from a multilevel and socioecological concept.

Such a perspective highlights the mutual relationship that might favor children to be more physically active to better support their potential development. This goal can be reached by analyzing and manipulating environmental affordances. Altered environmental contexts differently increase specific factors or the quality of motor competence.

According to this, a musical environment can promote optimal development of motor creativity through transdisciplinary training such as music and rhythmic exercises integration. Based on the ecological model, social and environmental interactions are integrated into physical literacy through a holistic approach to individual development.

Future research should also consider other variables, such as the effects of different school disciplines (e.g., art education and bilingualism) on motor creativity and motor competence. Such an investigation would provide a more straightforward overview of the magnitude of didactic enrichment due to each discipline. A transdisciplinary education system may benefit from it, emphasizing knowledge transferability while guiding the competent teacher in designing their physical education lessons in relation to the environmental constraints, health and anthropometric factors (as moderators of physical activity), as well as perceived experience (mediators of physical activity).

Summing up, only an integrated and holistic analysis can clarify how different processes, based on instructive and formative scholastic and extra-scholastic curricula, can interact and influence reciprocally to respond to a more complex and evolving society.

## Figures and Tables

**Table 1:** Participants’ characteristics (elementary and middle school students).

		Elementary-School					Middle-School				
EP	Sex	n	Age (years)	Weight (kg)	Height (cm)	BMI (kg/m^2^)	n	Age (years)	Weight (kg)	Height (cm)	BMI (kg/m^2^)
M-O	M	18	8.4 ± 0.9	32.3 ± 6.3	1.4 ± 0.1	17.2 ± 2.2	8	11.4 ± 4.1	47.8 ± 25.7	1.6 ± 0.9	18.5 ± 1.2
	F	14	7.1 ± 0.6	30.0 ± 10.5	1.3 ± 0.4	17.4 ± 2.6	18	11.8 ± 3.9	47.8 ± 21.8	1.6 ± 0.7	19.0 ± 2.0
C	M	30	7.0 ± 2.6	28.5 ± 12.6	1.3 ± 0.5	17.2 ± 2.0	24	12.3 ± 2.7	52.3 ± 21.7	1.6 ± 0.6	19.7 ± 2.6
	F	34	7.5 ± 2.5	28.6 ± 13.2	1.3 ± 0.6	17.3 ± 2.7	17	12.4 ± 3.1	48.3 ± 23.4	1.6 ± 0.8	18.9 ± 2.2

(EP) Educational plans. (M-O) Music-oriented. (C) Conventional. (BMI) Body mass index.

**Table 2:** Results of testing sessions by age, sex, and weight status.

Variable	EP	Age		Sex		Weight Status	
		Elementary- School	Middle- School	Boys	Girls	Normal Weight	Overweight
Stambak’s test							
Score	M-O	31.9 ± 3.2 #	**36.8 ± 3.2** #	33.4 ± 4.8	34.7 ± 3.2	**34.7 ± 4.0** #	32.6 ± 3.9 #
	C	25.2 ± 5.8	**30.1 ± 4.7**	27.4 ± 5.9	26.8 ± 5.9	**27.1 ± 5.7**	27.0 ± 3.9
DMA tasks							
Locomotor ^a^	M-O	10.8 ± 2.0 #	**24.3 ± 5.9** #	16.0 ± 9.2 #	17.5 ± 6.8 #	**18.2 ± 8.1** #	11.1 ± 3.4 #
	C	9.1 ± 5.7	**14.1 ± 9.6**	11.2 ± 8.1	10.8 ± 7.7	**13.0 ± 8.4**	7.7 ± 5.5
Stability ^a^	M-O	4.5 ± 5.0 #	**25.7 ± 7.4** #	11.2 ± 11.2 #	16.2 ± 12.8 #	**15.9 ± 12.0** #	5.8 ± 10.3 #
	C	3.4 ± 4.2	**12.7 ± 11.2**	7.0 ± 8.7	6.9 ± 9.2	**9.4 ± 10.3**	2.9 ± 2.8
Manipulative	M-O	9.6 ± 5.3 #	**16.6 ± 8.2** #	10.9 ± 8.3 #	**14.3 ± 6.7** #	**13.6 ± 7.5** #	9.0 ± 6.9 #
	C	5.9 ± 4.2	**11.9 ± 7.4**	7.8 ± 6.1	**8.9 ± 6.6**	**9.5 ± 6.8**	6.3 ± 5.1
KTK							
Walking backwards ^b^	M-O	**90.7 ± 14.1**	79.9 ± 17.0	88.7 ± 12.9	84.6 ± 18.3	**85.0 ± 16.5**	89.5 ± 15.2
	C	**88.5 ± 14.0**	83.3 ± 20.7	81.8 ± 16.5	91.9 ± 16.1	**87.3 ± 17.2**	84.9 ± 12.7
Hopping for height ^b^	M-O	93.6 ± 14.5	83.7 ± 19.1	**98.5 ± 13.7**	82.4 ± 16.2	**88.9 ± 19.3**	91.4 ± 10.9
	C	88.6 ± 15.9	88.5 ± 20.0	**88.8 ± 15.9**	88.3 ± 19.4	**87.1 ± 16.4**	91.4 ± 10.8
Jumping laterally ^a^	M-O	**102.7 ± 17.5**	86.0 ± 16.4	**105.7 ± 14.6**	88.1 ± 18.3	**92.5 ± 18.2**	103.7 ± 18.2
	C	**95.5 ± 15.1**	93.1 ± 12.9	**98.9 ± 11.2**	89.6 ± 15.8	**92.3 ± 15.0**	99.3 ± 17.0
Moving sideways	M-O	**92.3 ± 14.6**	75.8 ± 12.5	88.6 ± 15.3	83.4 ± 16.2	**83.1 ± 14.3**	91.4 ± 17.9
	C	**91.8 ± 15.7**	81.3 ± 20.3	86.7 ± 18.4	88.8 ± 18.3	**87.5 ± 18.1**	88.1 ± 16.2
Motor Quotient ^b^	M-O	**93.3 ± 14.5**	75.9 ± 19.1	**93.3 ± 14.0**	80.6 ± 19.8	**82.8 ± 18.9**	94.1 ± 15.0
	C	**88.5 ± 14.0**	81.3 ± 16.3	**85.5 ± 13.5**	86.1 ± 17.3	**84.6 ± 15.1**	88.0 ± 11.90
Multistage Fitness test							
Distance (m)	M-O	426.9 ± 119.3	**734.6 ± 341.2**	626.2 ± 332.9	515 ± 238.4	**592.7 ± 315.0**	497.6 ± 198.7
	C	346.6 ± 116.9	**754.6 ± 315.2**	537.0 ± 276.5	472.9 ± 311	**504.2 ± 313.8**	509.7 ± 204.7

Data are expressed in arbitrary units (AU) for Körperkoordinationstest Für Kinder (KTK), Divergent Movement Ability (DMA) tasks, and Stambak’s test. Note: EP = education plan, M-O = music-oriented, C = conventional. ^a^ Significant (age × education plan) interaction; ^b^ Significant (sex × education plan) interaction. Text in bold refers to the overall significant main effect for age, sex, and weight status, indicating the best result between the compared data (by column). # Significant main effect of education (M-O vs. C).

## Data Availability

The data presented in this study are available on request from the corresponding author.
